# Masitinib for mild-to-moderate Alzheimer’s disease: results from a randomized, placebo-controlled, phase 3, clinical trial

**DOI:** 10.1186/s13195-023-01169-x

**Published:** 2023-02-28

**Authors:** Bruno Dubois, Jesús López-Arrieta, Stanley Lipschitz, Doskas Triantafyllos, Luiza Spiru, Svitlana Moroz, Olena Venger, Patrick Vermersch, Alain Moussy, Colin D. Mansfield, Olivier Hermine, Magda Tsolaki, for the AB09004 Study Group Investigators

**Affiliations:** 1 Alzheimer Research Center IM2A, Salpêtrière Hospital, AP-HP, Sorbonne University, Paris, France; 2 Cantoblanco Memory Clinic, Geriatric Department, Hospital Cantoblanco, Madrid, Spain; 3 The Dr Stanley Lipschitz Clinic Inc, Rosebank, Johannesburg, South Africa; 4 Neurological Department, Athens Naval Hospital, Athens, Greece; 5 Carol Davila University of Medicine and Pharmacy, The Excellence Clinic of Geriatrics, Gerontology and Old Age Psychiatry, Bucharest, Romania; 6 The Excellence Memory Center and Longevity Medicine, “Ana Aslan” International Foundation, Bucharest, Romania; 7 Psychosomatic Center Based on Psychoneurology Department of Communal Enterprise ‘Dnipropetrovsk Regional Clinical Hospital named after I.I. Mechnikov’, Dnipropetrovsk Regional Council, Dnipro, Ukraine; 8 Department Psychiatry, Narcology and Medical Psychology I. Horbachevsky Ternopil National Medical University, Ternopil, Ukraine; 9 Univ. Lille, UMR Inserm U1172, CHU Lille, FHU Precise, F-59000 Lille, France; 10 AB Science, Paris, France; 11 Imagine Institute, INSERM UMR 1163, University of Paris, Laboratory of Cellular and Molecular Mechanisms of Hematological Disorders and Therapeutic Implication, Hôpital Necker, Paris, France; 12 Department of Hematology, Necker Hospital, Assistance Publique Hôpitaux de Paris, Paris, France; 13 First Department of Neurology, School of Medicine, Faculty of Health Sciences, Aristotle University of Thessaloniki, Thessaloniki, Macedonia Greece

**Keywords:** Alzheimer’s disease, Mast cells, Microglia, Tyrosine kinase inhibitor

## Abstract

**Background:**

Masitinib is an orally administered tyrosine kinase inhibitor that targets activated cells of the neuroimmune system (mast cells and microglia). Study AB09004 evaluated masitinib as an adjunct to cholinesterase inhibitor and/or memantine in patients with mild-to-moderate dementia due to probable Alzheimer’s disease (AD).

**Methods:**

Study AB09004 was a randomized, double-blind, two parallel-group (four-arm), placebo-controlled trial. Patients aged ≥50 years, with clinical diagnosis of mild-to-moderate probable AD and a Mini-Mental State Examination (MMSE) score of 12–25 were randomized (1:1) to receive masitinib 4.5 mg/kg/day (administered orally as two intakes) or placebo. A second, independent parallel group (distinct for statistical analysis and control arm), randomized patients (2:1) to masitinib at an initial dose of 4.5 mg/kg/day for 12 weeks that was then titrated to 6.0 mg/kg/day, or equivalent placebo. Multiple primary outcomes (each tested at a significance level of 2.5%) were least-squares mean change from baseline to week 24 in the Alzheimer’s Disease Assessment Scale - cognitive subscale (ADAS-cog), or the Alzheimer’s Disease Cooperative Study Activities of Daily Living Inventory scale (ADCS-ADL). Safety for each masitinib dose level was compared against a pooled placebo population.

**Results:**

Masitinib (4.5 mg/kg/day) (*n*=182) showed significant benefit over placebo (*n*=176) according to the primary endpoint of ADAS-cog, −1.46 (95% CI [−2.46, −0.45]) (representing an overall improvement in cognition) versus 0.69 (95% CI [−0.36, 1.75]) (representing increased cognitive deterioration), respectively, with a significant between-group difference of −2.15 (97.5% CI [−3.48, −0.81]); *p*<0.001. For the ADCS-ADL primary endpoint, the between-group difference was 1.82 (97.5% CI [−0.15, 3.79]); *p*=0.038 (i.e., 1.01 (95% CI [−0.48, 2.50]) (representing an overall functional improvement) versus −0.81 (95% CI [−2.36, 0.74]) (representing increased functional deterioration), respectively). Safety was consistent with masitinib’s known profile (maculo-papular rash, neutropenia, hypoalbuminemia). Efficacy results from the independent parallel group of titrated masitinib 6.0 mg/kg/day versus placebo (*n*=186 and 91 patients, respectively) were inconclusive and no new safety signal was observed.

**Conclusions:**

Masitinib (4.5 mg/kg/day) may benefit people with mild-to-moderate AD. A confirmatory study has been initiated to substantiate these data.

**Trial registration:**

EudraCT: 2010-021218-50. ClinicalTrials.gov: NCT01872598

**Supplementary Information:**

The online version contains supplementary material available at 10.1186/s13195-023-01169-x.

## Background

Alzheimer’s disease (AD) is a progressive neurodegenerative disease and the main cause of dementia, with an estimated prevalence of 50 million people worldwide that is projected to triple by 2050 [1]. The primary neuropathologic features of AD are the presence of extracellular β-amyloid (Aβ) and intracellular hyperphosphorylated tau deposits, which are linked through the amyloid cascade hypothesis [2]. Despite decades of extensive research, the majority of human trials (predominantly testing amyloid-based therapeutics) have failed to demonstrate clinical efficacy [3, 4]. This underscores a need for innovative, non-amyloid-based approaches, including therapies that modulate the neuroimmune response in AD, which has been implicated in the pathophysiology of the disease [4–8].

Masitinib is an oral tyrosine kinase inhibitor that has demonstrated neuroprotective action in neurodegenerative diseases via inhibition of mast cell and microglia/macrophage activity, and which is capable of accumulating within the central nervous system (CNS) at a therapeutically relevant concentration [9–12]. There is a growing body of evidence implicating mast cells and microglia (types of innate immune cells that are present in the CNS), with the pathophysiology of AD [13–26]. Masitinib has been shown to restore normal spatial learning performance and promote recovery of synaptic markers in a mouse model of AD, with its synapto-protective action being directly linked to mast cell inhibition [27]. Previously, a small phase 2 trial showed that masitinib slows progression in mild-to-moderate AD patients [28]. Here, we report findings from study AB09004, the first large randomized trial targeting activated neuroimmune cells for treatment of mild-to-moderate AD [29].

## Methods

### Study oversight

Study AB09004 (NCT01872598) was overseen by an Independent Data Monitoring Committee (IDMC). The trial protocol was approved by the appropriate Independent Ethics Committee/Institutional Review Board of all participating sites and all subjects provided informed consent. The sponsor (AB Science) participated in the design, conduct, management, and reporting of the study. Data was collected and analyzed in conjunction with independent steering committee members and authors, who contributed to manuscript draft revisions, provided critical comment, and approved submission for publication.

**Fig. 1:**
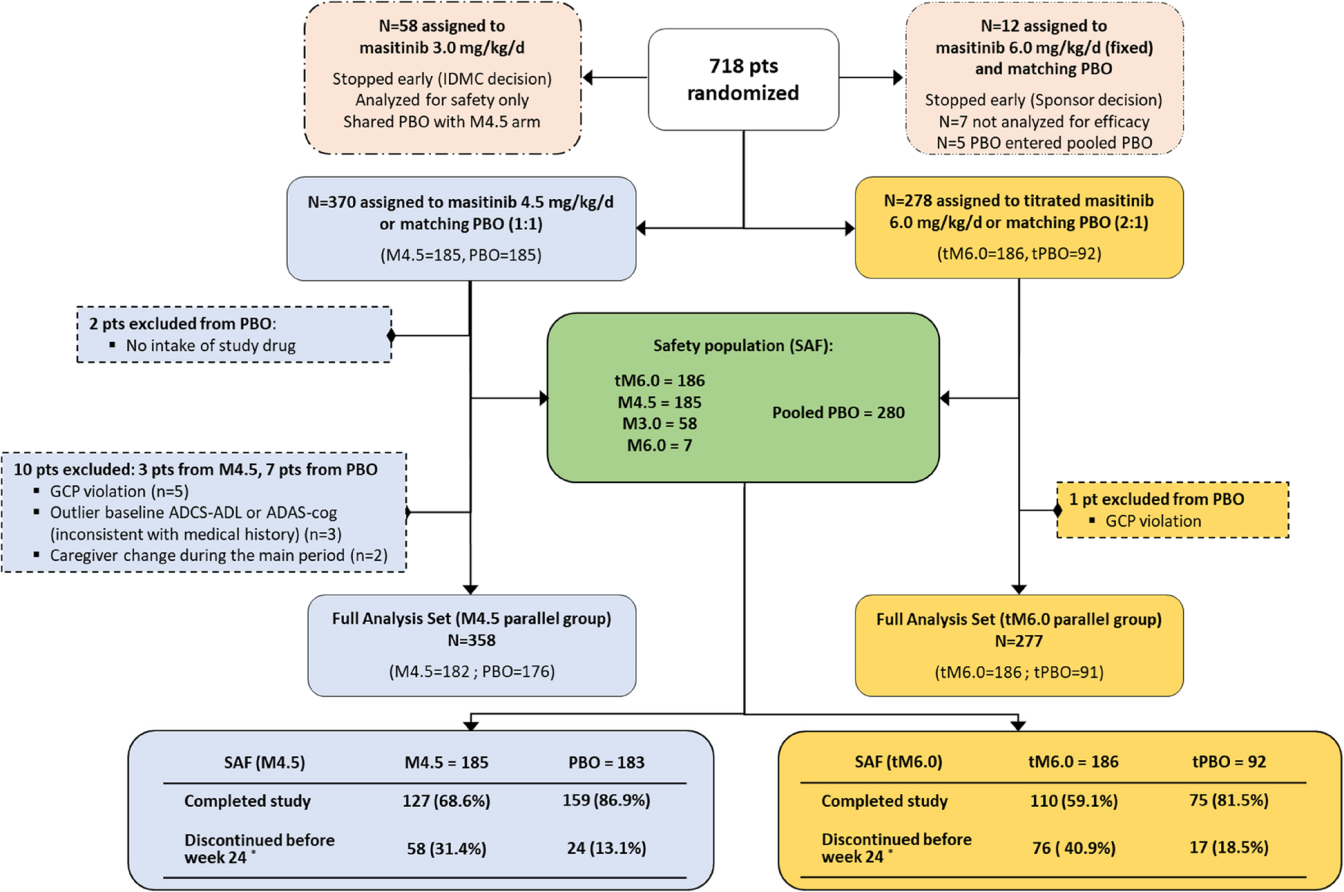
Patient flow diagram, detailing patient disposition of the masitinib 4.5 mg/kg/day and titrated masitinib 6.0 mg/kg/day parallel groups. PBO, placebo; M4.5, masitinib 4.5 mg/kg/day; tPBO, placebo treatment-arm from the titrated dose parallel group; tM6.0, masitinib treatment-arm from the titrated dose parallel group; ITT, intention-to-treat population; SAF, safety population; FAS, full analysis database; GCP, Good Clinical Practice; ADAS-cog, Alzheimer’s Disease Assessment Scale - cognitive subscale; ADCS-ADL, Alzheimer’s Disease Cooperative Study Activities of Daily Living Inventory scale. * See eTable 1 of the Supplemental Information for a summary of reasons for discontinuation before week 24

Protocol amendments were implemented during the study following approval by the aforementioned committees. Briefly, study AB09004 initially planned to enroll patients into placebo or masitinib 6 mg/kg/day treatment-arms (1:1); however, a comprehensive, global safety analysis of all masitinib non-oncology clinical trials (not including the current AB09004 study) revealed that masitinib starting doses of 3 or 4.5 mg/kg/day had an incidence of severe adverse events similar to placebo, whereas a starting dose of 6 mg/kg/day showed increased frequency of certain events with respect to placebo (e.g., neutropenia and skin toxicity). Moreover, related analysis also revealed that starting doses of 3 or 4.5 mg/kg/day titrated to 6 mg/kg/day improved tolerability and minimized discontinuations during the first 3 months of treatment. Protocol amendments, with the objective to improve the benefit/risk balance, were therefore an unavoidable consequence of these developments. First, the 6 mg/kg/day (starting dose) treatment-arm was terminated (protocol version 5.0, May 2012, after recruitment of 12/718 (1.7%) patients) and replaced with a placebo versus masitinib at 4.5 or 3.0 mg/kg/day (1:1:1) design (administered orally as two daily intakes). The low-dose 3.0 mg/kg/day masitinib treatment-arm was later terminated (following recommendation from the IDMC for reasons not based on safety or futility), effectively collapsing this parallel group into a (1:1) comparison of masitinib 4.5 mg/kg/day versus placebo (protocol version 9.0, November 2015, after 25% (180/718) of randomized patients could have reached the 24-week timepoint) (Fig. 1). Second, an independent parallel group was added to the study in which patients were randomly assigned to receive a placebo or masitinib as a titrated treatment regimen, i.e., an initial dose of 4.5 mg/kg/day for 12 weeks that was then titrated to a planned dose of 6.0 mg/kg/day (as per protocol version 6.0; August 2013).

Another notable amendment concerned the primary efficacy analysis, originally defined as co-primary endpoints of the Alzheimer’s Disease Assessment Scale - cognitive subscale (ADAS-cog) and the Alzheimer’s Disease Cooperative Study Activities of Daily Living Inventory scale (ADCS-ADL) at week 24 (significance level of 5%), which was modified to multiple primary endpoints of ADAS-cog or ADCS-ADL at week 24 (based on fallback procedure with a significance level of 2.5%) (protocol version 10.0, December 2017, after 81% (580/718) of randomized patients could have reached the 24-week timepoint). This was done to give cognitive and functional outcomes equal importance, with autonomous assessment of each.

### Study design

Study AB09004 was an international, double-blind, multicenter, phase 3, randomized, placebo-controlled trial over a 24-week treatment period. Following the aforementioned protocol amendments, two prospectively declared parallel groups, distinct in matters of statistical analysis and control arm (i.e., effectively run as separate studies), were assessed.

Patients were centrally randomized using an interactive web-response system according to a computer-generated assignment schedule and minimization method with covariates of ADAS-cog total score, ADCS-ADL total score, severity of baseline disease (Mini-Mental State Examination (MMSE) 21–25 versus 12–20), and age at baseline (50–79 versus ≥80). Patients and study staff were masked to treatment assignment for the study duration.

### Assessments

During the 24-week assessment period, clinical efficacy was measured at week-0, week-8, week-12, and week-24 according to the following instruments: 11-item ADAS-cog (scores range from 0 to 70, with higher scores indicating worse dementia) [30]; 23-item ADCS-ADL (scores from 0 to 78, with lower scores indicating worse function) [31]; Clinician’s Interview-Based Impression of Change plus Caregiver Input (CIBIC-plus), a seven-point categorical rating scale ranging from 1 (marked improved) to 7 (markedly worse) compared with baseline [32]; MMSE (scores from 0 to 30, with lower scores indicating poorer cognitive performance) [33]; and Clinical Dementia Rating Scale (CDR, scores from 0 to 18, with higher scores indicating worse dementia) [34]. All analyses and reporting procedures were performed using SAS version 9.4 (SAS Institute. Cary, NC).

Patients were monitored for safety from the date of informed consent until 28 days after discontinuing the study drug. Safety for each masitinib dose level was compared against a pooled placebo population and expressed in terms of incidence rate ratio (IRR) (i.e., the incidence rate of masitinib divided by that of pooled placebo). Adverse events were coded according to the MedDRA dictionary version 20.0.

### Patient population

Patients were eligible for enrollment if they met standard clinical criteria for dementia that was probably due to AD (biomarker tests were not required for patient inclusion) [35, 36], had a baseline MMSE score of 12 to 25 (corresponding to mild or moderate dementia), had been treated with a cholinesterase inhibitor, memantine, or both, (representing the standard of care) for a minimum of 6 months prior to screening, and were at least 50 years old. Exclusion criteria included any other cause of dementia not due to AD, severe forms of delusions or delirium, presence of infection, evidence/history of significant psychiatric disorder, and treatment with registered or putative cognitive/memory enhancer or disease modifier (other than donepezil, galantamine, rivastigmine or memantine).

### Outcomes and statistical analysis

The primary analysis was absolute change (δ) from baseline on ADAS-cog or ADCS-ADL over 24 weeks (positive δADAS-cog indicating worsening dementia while negative δADCS-ADL indicates worsening function), with treatment effect being demonstrated by a significant between-group difference (Δ, masitinib versus placebo) on at least one of the multiple primary endpoints, with a significance level of 0.025. A negative ΔADAS-cog value or positive ΔADCS-ADL value favors masitinib. Results were calculated using a model of analysis of covariance (ANCOVA) adjusted on the aforementioned stratification variables and expressed as least-squares mean (LSM) change from baseline with corresponding 97.5% two-sided confidence intervals (CI) and statistical test P-value. Primary efficacy analysis was done according to a full analysis dataset (FAS) in which patients received at least one dose of the trial regimen and whose scores had not been potentially influenced by any source of strong bias during the 24-week assessment period (according to predefined rules and validated by the IDMC prior to unblinding; see Supplemental Information). When individual subscores were missing, they were imputed via last observation carried forward methodology to enable the computation of a total score. Missing total scores due to patient discontinuation were imputed via clustering methodology based on treatment group assignment and the randomization stratification factors of MMSE and age (thereby, defining groups that are approximately homogeneous with respect to the target variable). This imputation method calculates the average disease progression until week 24 among patients with complete data from the same cluster, then imputes this incremental trend to give an estimate of the individual’s total score at week 24 (i.e., single mean imputation within classes) [37].

Sensitivity analyses and secondary endpoints were tested at the 0.05 significance level. Consistency of the primary analysis was tested using predefined sensitivity analyses including multiple imputation and the conservative jump-to-reference method. This latter approach assumes that patients who discontinue treatment for lack of efficacy or safety will no longer benefit from it in the future, and thus will tend to have outcomes similar to those in the control group [38]. A key secondary endpoint was analysis of clinical response (logistic regression model with logit as link function), wherein a positive response was defined as a decrease from baseline at week 24 in ADAS-cog of ≥4 [39], without deterioration in ADCS-ADL or worsening in the CIBIC-plus scale (≥5). Other secondary endpoints included: MMSE and CDR (assessed according to change from baseline on timepoints of week 8, week 12, and week 24 using a mixed model of repeated measures methodology), and assessment of CIBIC-plus improvement (i.e., a score of 1–3) or worsening (i.e., a score of 5–7) at week-24 (chi-square test).

Based on phase 2 (AB04024) study data, we estimated that change in ADCS-ADL at week 24 would be −0.6 (±9.0) for placebo, +2.5 (±9.0) for masitinib 4.5 mg/kg/day, and +3.2 (±9.0) for masitinib 6.0 mg/kg/day, while change in ADAS-cog at week 24 would be +2.0 (±7.0) for placebo, −0.4 (±7.0) for masitinib 4.5 mg/kg/day, and −1.0 (±7.0) for masitinib 6.0 mg/kg/day. Detection of this difference, with a two-sided 0.05 significance level and a power of 80%, would require a minimum sample size of 300 patients for the masitinib 4.5 mg/kg/day parallel group (150 per treatment-arm), and a total of 225 patients for the titrated masitinib 6.0 mg/kg/day parallel group (150 masitinib versus 75 placebo).

## Results

### Patients

From February 2012 to September 2018 (database lock in November 2020), a total of 718 patients from 119 hospital clinics and specialized AD centers in 20 countries were randomized to study AB09004 (see Supplemental Information for list of countries); 370 in the masitinib 4.5 mg/kg/day parallel group (185 masitinib versus 185 placebo), and 278 in the titrated masitinib 6.0 mg/kg/day parallel group (186 masitinib versus 92 placebo). Additionally, 58 masitinib patients were in the terminated masitinib 3.0 mg/kg/day treatment-arm and 12 patients were in the terminated 6.0 mg/kg/day treatment-arm (7 masitinib versus 5 placebo). The safety population comprised all patients that received at least one dose of study medication with each masitinib dose level compared against a pooled placebo population (*n*=280) (Fig. 1).

Considering the Full Analysis Dataset (FAS) of the masitinib 4.5 mg/kg/day parallel-group, 12 patients were excluded from its associated intention-to-treat (ITT) population (3 and 9 from the masitinib and placebo treatment-arms, respectively; see Supplemental Information). A summary of patient baseline characteristics and disposition are described in Table 1 and Fig. 1, respectively. Patients from each treatment-arm had a median age of 73 years that was evenly distributed across the range of 50–88 years old. Baseline median MMSE, ADAS-cog, and ADCS-ADL scores were also well-balanced. Premature discontinuation before week 24 was higher for masitinib-treated patients at 34.5% (148/429) as compared with the pooled placebo group at 14.6% (41/282), with the most frequent reasons being treatment related non-fatal adverse events, and patients request or withdrawal of consent (eTable 1 of the Supplemental Information).

**Table 1:** Baseline patient characteristics of the masitinib 4.5 mg/kg/day and titrated masitinib 6.0 mg/kg/day parallel groups with respective placebo-control arms (FAS population)

		Masitinib 4.5 mg/kg/day parallel group		Titrated masitinib 6.0 mg/kg/day parallel group	
		M4.5 (***n***=182)	PBO (***n***=176)	tM6.0 (***n***=182)	tPBO (***n***=91)
**Gender**	Female [*n* (%)]	114 (62.6%)	98 (55.7%)	118 (63.4%)	57 (62.6%)
**Age (years)**	Mean (±SD)	71.9 (±8.3)	71.7 (±8.2)	71.9 (±8.3)	71.2 (±8.1)
	Median	73.0	73.0	72.0	72.0
	Range (min–max)	50.0–86.0	50.0–88.0	50.0–88.0	51.0–87.0
	>=50–<60 [*n* (%)]	19 (10.4%)	17 (9.4%)	14 (7.5%)	10 (11.0%)
	>=60–<70 [*n* (%)]	44 (24.0%)	52 (28.9%)	54 (29.0%)	26 (28.6%)
	>=70–<80 [*n* (%)]	82 (44.8%)	76 (42.2%)	78 (41.9%)	41 (45.1%)
	≥ 80 [*n* (%)]	38 (20.8%)	35 (19.4%)	40 (21.5%)	14 (15.4%)
**MMSE**	Mean (±SD)	18.8 (±3.7)	18.6 (±3.8)	18.8 (±3.6)	18.7 (±3.7)
	Median	19.0	19.0	19.0	19.0
	MMSE [12–20] [*n* (%)]	119 (65.4%)	115 (65.3%)	123 (66.1%)	57 (62.6%)
	MMSE [21–25] [*n* (%)]	63 (34.6%)	61 (34.7%)	63 (33.9%)	34 (37.4%)
**ADCS-ADL**	Mean (±SD)	51.8 (±15.1)	51.4 (±15.0)	52.4 (±14.8)	53.2 (±13.7)
	Median	55.0	53.5	54.0	57.0
	Range (min–max)	13.0–78.0	4.0–77.0	9.0–78.0	18.0–77.0
**ADAS-cog**	Mean (±SD)	26.1 (±10.1)	25.9 (±9.7)	24.9 (±10.1)	26.2 (±10.6)
	Median	25.5	24.8	24.3	24.3
	Range (min–max)	7.2–54.3	9.2–53.0	4.7–57.3	6.8–51.7
**CDR**	Mean (±SD)	1.3 (±0.6)	1.2 (±0.8)	1.2 (±0.6)	1.1 (±0.6)
	Median	1	1	1	1
	Range (min–max)	0.5–3	0.5–3	0–3	0.5–2

*M4.5* masitinib treatment-arm from the masitinib 4.5 mg/kg/day parallel group, *PBO* placebo treatment-arm from the masitinib 4.5 mg/kg/day parallel group, *tM6.0* masitinib treatment-arm from the titrated 6.0 mg/kg/day parallel group, *tPBO* placebo treatment-arm from the titrated 6.0 mg/kg/day parallel group, *MMSE* Mini-Mental State Examination, *ADAS-cog* Alzheimer’s Disease Assessment Scale - cognitive subscale, *ADCS-ADL* Alzheimer’s Disease Cooperative Study Activities of Daily Living Inventory scale, *CDR* Clinical Dementia Rating Scale, *SD* standard deviation

Considering the titrated masitinib 6.0 mg/kg/day parallel group FAS, one placebo patient was excluded from the ITT population (Fig. 1). Baseline median MMSE and ADAS-cog scores were balanced between treatment-arms, as was the median age at 72 years old; however, the masitinib arm had a higher proportion of over 80-year-olds compared with placebo (21.5% versus 15.4%) and a lower median ADCS-ADL score (54.0 versus 57.0) (Table 1).

### Primary efficacy analysis

Masitinib (4.5 mg/kg/day) showed significant benefit relative to placebo over 24 weeks on the endpoint of ADAS-cog, with a δADAS-cog of −1.46 (representing an overall improvement in cognition) versus +0.69 (representing increased cognitive deterioration), respectively, and corresponding ΔADAS-cog of −2.15 (97.5%CI [−3.48, −0.81]); *p*<0.001. The primary endpoint of ADCS-ADL numerically favored masitinib with a δADCS-ADL of +1.01 (representing an overall functional improvement) versus -0.81 for placebo (representing increased functional deterioration), respectively, giving a nonsignificant ΔADCS-ADL of +1.82 (97.5%CI [(−0.15, 3.79]); *p*=0.038.

Jump-to-reference and multiple imputation sensitivity analyses confirmed the study’s primary objective was achieved for the primary endpoint of ADAS-cog, with a significant ΔADAS-cog of −1.86 (95%CI [(−3.03, −0.69]; *p*=0.002) and −2.04 (95%CI [(−3.41, −0.67]; *p*=0.004), respectively. Sensitivity analysis based on the ITT population of the masitinib 4.5 mg/kg/day parallel group further corroborate these findings (indicating that no bias in favor of treatment had been introduced by exclusion of patients for the FAS population), with a significant ΔADAS-cog of −2.08 (95%CI [(−3.22, −0.94]; *p*<0.001) (Table 2). An additional post hoc analysis according to mixed model for repeated measures (MMRM) methodology also produced a significant ΔADAS-cog of −0.95 (95%CI [−1.89 −0.02]; *p*=0.046), while ADCS-ADL analysis under these conditions remained nonsignificant (eTable 2 in the Supplemental Information).

**Table 2:** Summary of efficacy results for primary analysis, sensitivity analyses, and secondary endpoints (responder analyses) for the masitinib 4.5 mg/kg/day and titrated masitinib 6.0 mg/kg/day parallel groups (FAS population)

M4.5 parallel group		PBO (***N***=176)	M4.5 (***N***=182)	Between group difference	***P*** value
	**Primary analysis**	**Change from baseline at week 24 (±SE)**		**LSM (97.5% CI)**	
	ADAS-Cog (primary endpoint)	0.69 (±0.54)	−1.46 (±0.51)	−2.15 [−3.48, −0.81]	<0.001
	ADCS-ADL (primary endpoint)	−0.81 (±0.79)	1.01 (±0.76)	1.82 [−0.15, 3.79]	0.038
	**Sensitivity analysis**	**Change from baseline at week 24 (±SE)**		**LSM (95% CI)**	
	ADAS-Cog (JTR analysis)	0.85 (±0.54)	−1.04 (±0.52)	−1.89 [−3.06, −0.72]	0.002
	ADCS-ADL (JTR analysis)	−0.90 (±0.79)	0.81 (±0.76)	1.71 [−0.01, 3.43]	0.051
	ADAS-Cog (MI analysis)	0.88 (±0.63)	−1.16 (±0.65)	−2.04 [−3.41, −0.67]	0.004
	ADCS-ADL (MI analysis)	−0.95 (±0.93)	0.77 (±0.90)	1.72 [−0.26, 3.70]	0.089
	ADAS-Cog (ITT analysis) ^a^	0.67 (±0.53)	−1.41 (±0.50)	−2.08 [−3.22, −0.94]	<0.001
	ADCS-ADL (ITT analysis) ^a^	−0.06 (±0.83)	1.14 (±0.80)	1.20 [−0.60, 3.00]	0.192
	**Responder analyses (secondary endpoints)**	**Response rate,** ***n*** **(%)**		**Odds Ratio (95% CI)**	
	Clinical response rate	23 (13.1%)	41 (22.5%)	1.96 [1.11, 3.46]	0.020
	CIBIC-plus improvement	36 (20.5%)	47 (25.8%)	1.71 [1.02, 2.85]	0.040
	CIBIC-plus worsening	37 (21.0%)	23 (12.6%)	0.64 [0.36, 1.14]	0.127
**tM6.0 parallel group**		**tPBO (*N*=91)**	**tM6.0 (*N*=186)**	**Between group difference**	***P*** **value**
	**Primary analysis**	**Change from baseline at week 24 (±SE)**		**LSM (97.5% CI)**	
	ADAS-Cog (primary endpoint)	0.25 (±0.60)	−0.18 (±0.47)	−0.43 [−1.81, 0.95]	0.483
	ADCS-ADL (primary endpoint)	0.37 (±0.81)	0.57 (±0.62)	0.20 [−1.64, 2.04]	0.807

Unless otherwise stated, this table summarizes data from patients in the masitinib 4.5 mg/kg/day parallel group full analysis dataset (PBO *n*=176; M4.5 *n*=182). All assessments were prespecified in the protocol or Statistical Analysis Plan prior to unblinding. *M4.5* masitinib treatment-arm from the masitinib 4.5 mg/kg/day parallel group, *PBO* placebo treatment-arm from the masitinib 4.5 mg/kg/day parallel group, *tM6.0* masitinib treatment-arm from the titrated 6.0 mg/kg/day parallel group, tPBO placebo treatment-arm from the titrated 6.0 mg/kg/day parallel group, *LSM* least-squares mean, *JTR* jump-to-reference, *MI* multiple imputation, *ITT* intention-to-treat population, *ADAS-cog* Alzheimer’s Disease Assessment Scale. For ADAS-cog (scores range from 0 to 70) a positive change from baseline indicates worsening dementia and a negative between group difference (masitinib minus placebo) favors masitinib. *ADCS-ADL* Alzheimer’s Disease Cooperative Study Activities of Daily Living Inventory scale. For ADCS-ADL (scores range from 0 to 78) a negative change from baseline indicates worsening function and a positive between group difference (masitinib minus placebo) favors masitinib. CIBIC-plus: Clinician’s Interview-Based Impression of Change plus Caregiver Input (scores of 1-3 correspond with improvement, scores of 5-7 correspond with worsening compared with baseline). Clinical response defined as decrease from baseline at week 24 in ADAS-cog of ≥4, without deterioration in ADCS-ADL or worsening in the CIBIC-plus scale. SE: Standard error. CI: Confidence Interval at 97.5% for primary endpoint and at 95% for sensitivity analyses. Sensitivity and secondary endpoint analyses on the titrated masitinib 6.0 mg/kg/day dataset were not performed because, in accordance to protocol, the primary endpoint was not met for this parallel group. ^a^ Sensitivity analysis performed on the ITT population (PBO *n*=185; M4.5 *n*=186)

Results from the titrated masitinib dose of 6.0 mg/kg/day parallel group did not show any significant difference between the masitinib and placebo treatment arms for either ADAS-cog or ADCS-ADL; ΔADAS-cog was −0.43 (97.5%CI [(−1.81, 0.95]), *p*=0.483; while ΔADCS-ADL was +0.20 (97.5%CI [(−1.64, 2.04]), *p*=0.807 (Table 2).

### Secondary endpoint analyses

Considering the masitinib 4.5 mg/kg/day parallel-group, masitinib-treated patients showed a significantly increased probability of cognitive improvement at week 24 relative to placebo, with a clinical responder rate of 22.5% versus 13%, respectively (odds ratio 1.96 (95%CI [1.11, 3.46]); *p*=0.020) (Table 2). Significance was also reached for analysis of CIBIC-Plus improvement at week 24 (odds ratio 1.71 (95%CI [1.02, 2.85]); *p*=0.040). There was no discernable effect between treatment-arms at week 24 for the outcomes of CDR (−0.025 (95%CI [−0.146, 0.095]) and MMSE (0.226 (95%CI [−0.4634, 0.9164]). In accordance to protocol, secondary endpoint analyses on the titrated masitinib 6.0 mg/kg/day dataset were not performed because the primary endpoint was not met for this parallel group.

### Safety analysis

The incidence of treatment-emergent adverse events (AE) for masitinib (4.5 mg/kg/day), titrated masitinib (6.0 mg/kg/day), and pooled placebo was 87% (161/185), 86% (160/186), and 77.5% (217/280), respectively (Table 3). The corresponding IRRs relative to pooled placebo were 1.1 for both masitinib parallel groups. The incidence of severe AE was 26.5% (49/185, IRR=1.4) for masitinib (4.5 mg/kg/day), 25.3% (47/186, IRR=1.3) for titrated masitinib (6.0 mg/kg/day), and 19.3% (54/280) for pooled placebo, while the incidence of non-fatal serious AE (SAE) was 13% (24/185, IRR=2.4), 13.4% (25/186, IRR=2.5), and 5.4% (15/280), respectively. Analysis of common severe AEs (i.e., ≥1% difference in incidence between masitinib and pooled placebo or with an IRR of ≥2) showed that an increased incidence of neutropenia and various other laboratory assessments for masitinib relative to placebo accounted for the difference in overall incidence (eTable 3 of the Supplemental Information). The most common SAE (MedDRA preferred terms) for masitinib relative to pooled placebo were neutropenia (all masitinib doses), pneumonia (3 patients for masitinib 4.5 mg/kg/day), and Stevens-Johnson syndrome (3 patients for titrated masitinib 6.0 mg/kg/day) (eTable 4 in the Supplemental Information). None of the Stevens-Johnson syndrome events were life-threatening and upon further analysis by dermatology experts, each case was considered as inconclusive (i.e., possible SJS). There was one death in each of these treatment-arms, none of which were treatment-related.

**Table 3:** Safety summary of treatment-emergent adverse events over the 24-week treatment period and corresponding incidence rate ratios (Safety population)

Pts with ≥1 event; % (n)	M3.0 (***N***=58)	IRR_**[M3.0]**_	M4.5 (***N***=185)	IRR_**[M4.5]**_	tM6.0 (***N***=186)	IRR_**[tM6.0]**_	Pooled PBO (***N***=280)
^a^ **AE (any grade)**	91.4% (53)	1.2	87.0% (161)	1.1	86.0% (160)	1.1	77.5% (217)
**AE leading to death**	0	0	0.5% (1)	1.3	0.5% (1)	1.3	0.4% (1)
**Serious AE (non-fatal)**	10.3% (6)	1.9	13.0% (24)	2.4	13.4% (25)	2.5	5.4% (15)
**Severe AE (grades 3 or 4)**	15.5% (9)	0.8	26.5% (49)	1.4	25.3% (47)	1.3	19.3% (54)
**TEAE leading to permanent discontinuation**	24.1% (14)	4.8	15.7% (29)	3.1	24.2% (45)	4.8	5.0% (14)
**TRAE leading to permanent discontinuation**	12.1% (7)	3.1	12.4% (23)	3.2	21.5% (40)	5.5	3.9% (11)
^*b*^ *Mild / Moderate*	*5.2% (3)*		*7.0% (13)*		*14.0% (26)*		*3.9% (11)*
^*b*^*Severe*	*6.9% (4)*		*4.7% (9)*		*6.5% (12)*		*0*

^a^ Adverse events (AE) were recorded until 28 days after treatment interruption with any AE not resolved at the death of the patients recorded as an AE leading to death. TEAE: Treatment-emergent adverse event leading to permanent discontinuation excluding death. *TRAE* treatment-related adverse event leading to permanent discontinuation excluding death. ^b^ TRAE according to severity. *M3.0* masitinib treatment-arm from the masitinib 3.0 mg/kg/day parallel group, *M4.5* masitinib treatment-arm from the masitinib 4.5 mg/kg/day parallel group, *tM6.0* masitinib treatment-arm from the titrated 6.0 mg/kg/day parallel group, *PBO* placebo, *IRR* incidence rate ratio for given masitinib cohort as compared with pooled placebo cohort (estimated as incidence rates of masitinib divided by pooled placebo)

## Discussion

Masitinib was administered as an adjunct therapy to standard of care in patients with mild to moderate dementia due to probable AD. After 24 weeks of treatment, masitinib (4.5 mg/kg/day) significantly slowed cognitive deterioration (as measured by the primary endpoint of ADAS-cog), with acceptable safety. This positive outcome was supported by convergence in ADAS-cog sensitivity analyses (jump-to-reference and ITT), a statistically significant clinical response rate, and a nonsignificant trend towards improved overall function relative to placebo (as measured by the second primary endpoint of ADCS-ADL). Conversely, results from the titrated masitinib 6.0 mg/kg/day parallel group did not demonstrate any treatment effect. One explanation of this divergent result is that the titrated 6.0 mg/kg/day parallel group placebo arm showed an atypical improvement over 24 weeks, as exemplified by the positive change from baseline in ADCS-ADL score (eFigure 1 in the Supplemental Information). This scenario is supported by a post hoc sensitivity analysis using the pooled placebo cohort (*n*=267), in which an expected worsening in ADAS-cog and ADCS-ADL is observed for placebo, with stable or improved score for the titrated masitinib 6.0 mg/kg/day cohort and a significant treatment effect (at an alpha level of 0.05) in terms of the ADAS-cog primary endpoint (eTable 5 in the Supplemental Information). Nevertheless, in the absence of evidence for a masitinib dose-dependent treatment effect (i.e., the change from baseline in ADAS-cog and ADCS-ADL was smaller for the titrated 6.0 mg/kg/day masitinib arm relative to the 4.5 mg/kg/day masitinib arm) and equivalent safety profiles, the recommended masitinib dose in terms of benefit/risk balance for future clinical development is 4.5 mg/kg/day.

Administering masitinib as an adjunct to cholinesterase inhibitor and/or memantine only slightly increased the overall incidence rate ratio of AEs with respect to placebo (regardless of severity). Regarding the higher rate of patient discontinuation due to treatment-related AE in the masitinib arm as compared with placebo, a large proportion of this (about 60%) was attributable to AEs of mild or moderate severity that can be efficiently managed by dose reduction or temporary interruption.

A limitation of our trial was the lack of fluid- and imaging-based biomarkers as evidence to support modification of underlying disease processes (e.g., cerebrospinal fluid tests measuring tau or measuring the brain volume using MRI scanning), or proof of target engagement (e.g., neuroinflammatory markers). Biomarker tests, as recommended by the IWG or by the NIA-AA diagnostic criteria [40, 41], were also not required for patient inclusion, which was based instead on standard clinical criteria for dementia that was probably AD [35, 36]. Regarding the previously mentioned protocol amendments, these had no impact on the study outcome. Termination of the masitinib 6.0 mg/kg/day (starting dose) and 3.0 mg/kg/day treatment-arms occurred early in the study and without consequence to analysis of the remaining arms; furthermore, the original co-primary endpoint definition (i.e., change from baseline at week 24 in both ADAS-cog and ADCS-ADL at an alpha level of 0.05) would also have returned a significant result for masitinib (4.5 mg/kg/day), had it been retained.

## Conclusions

Study AB09004 represents the first successful randomized, controlled, phase 3 trial in AD of a tyrosine kinase inhibitor, targeting innate immune cells. Given the known targets of masitinib, these positive clinical findings suggest that mast cells and/or macrophage/microglia are implicated in the pathophysiology of mild-to-moderate AD, possibly by switching the neuroimmune system from a neurotoxic state towards a neuroprotective state through remodeling of the neuronal microenvironment [8, 14].

In the absence of a consensus regarding the minimal clinically important change for ADAS-Cog [42, 43], the most appropriate comparison comes from benchmark ADAS-Cog benefit according to well-established AD therapies. Multiple approved drug treatments and dosages for AD have demonstrated a similar change in ADAS-Cog (approximately 2-point) to that reported for masitinib (4.5 mg/kg/day) and this value is also consistent with published recommendations [44–47]. The observed improvement in ADAS-Cog for masitinib (4.5 mg/kg/day) relative to control is therefore clinically meaningful, especially when considering its administration on a background of cholinesterase inhibitors and memantine, a significant clinical response rate (i.e., based on a criterion of ≥4-point improvement in ADAS-Cog), and manageable safety profile. Based on these results, masitinib (4.5 mg/kg/day) as an adjunct to cholinesterase inhibitor and/or memantine, could benefit patients with mild-to-moderate AD, and a confirmatory pivotal study, including biomarker outcomes, has been initiated.

## Supplementary Information

**Additional file 1: Supplemental methods.** Full Analysis Dataset (FAS) definition for primary efficacy analysis of the masitinib 4.5 mg/kg/day parallel group. **eTable 1.** Summary of reasons for discontinuation before week 24, based on information recorded on the case report form (CRF) end-of-study page (Safety dataset). **eTable 2.** Exploratory post-hoc analysis for the masitinib 4.5 mg/kg/day parallel group based on MMRM methodology (FAS population). **eTable 3.** Summary of most frequent severe adverse events for masitinib cohorts relative to pooled placebo cohort over the 24-week treatment period and corresponding incidence rate ratios (Safety dataset). **eTable 4.** Summary of most frequent non-fatal serious adverse events for masitinib cohorts relative to pooled placebo cohort over the 24-week treatment period and corresponding incidence rate ratios (Safety dataset). **eTable 5.** Additional pooled placebo sensitivity analysis for the titrated masitinib 6.0 mg/kg/day parallel group (FAS population). **eFigure 1.** Treatment effect on ADAS-cog and ADCS-ADL between week 0 and week 24 in the 4.5 mg/kg/day parallel group (left panel) and titrated 6.0 mg/kg/day parallel group (right panel).

## Data Availability

Masitinib is under clinical investigation and has not yet been approved in any sought-after indication by any health authority worldwide. As such, there is no plan for data-sharing at this point in time.

## Abbreviations

AD: Alzheimer’s disease
ADAS-cog: Alzheimer’s Disease Assessment Scale - cognitive subscale
ADCS-ADL: Alzheimer’s Disease Cooperative Study Activities of Daily Living Inventory scale
AE: Adverse event
ANCOVA: Model of analysis of covariance
CDR: Clinical Dementia Rating Scale
CI: Confidence intervals
CIBIC-plus: Clinician’s Interview-Based Impression of Change plus Caregiver Input
CNS: Central nervous system
FAS: Full analysis dataset
GCP: Good Clinical Practice
IDMC: Independent Data Monitoring Committee
IRR: Incidence rate ratio
ITT: Intention-to-treat
IWG: International Working Group
JTR: Jump-to-reference
LSM: Least-squares mean
M4.5: Masitinib 4.5 mg/kg/day
MI: Multiple imputation
MMRM: Mixed Model for Repeated Measures
MMSE: Mini-Mental State Examination
NIA-AA: National Institute on Aging and Alzheimer’s Association
PBO: Placebo
SAE: Serious adverse event
SAF: Safety population
SD: Standard deviation
SE: Standard error
TEAE: Treatment-emergent adverse event
tM6.0: Masitinib treatment-arm from titrated dose parallel group
tPBO: Placebo treatment-arm from titrated dose parallel group
TRAE: Treatment-related adverse event
ΔADAS-cog: Between-group difference (masitinib versus placebo) on ADAS-cog
δADAS-cog: Absolute change from baseline on ADAS-cog over 24 weeks
ΔADCS-ADL: Between-group difference (masitinib versus placebo) on ADCS-ADL
δADCS-ADL: Absolute change from baseline on ADCS-ADL over 24 weeks
