# Correction: Masitinib for mild-to-moderate Alzheimer’s disease: results from a randomized, placebo-controlled, phase 3, clinical trial

**DOI:** 10.1186/s13195-023-01230-9

**Published:** 2023-04-22

**Authors:** Bruno Dubois, Jesús López‑Arrieta, Stanley Lipschitz, Triantafyllos Doskas, Luiza Spiru, Svitlana Moroz, Olena Venger, Patrick Vermersch, Alain Moussy, Colin D. Mansfield, Olivier Hermine, Magda Tsolaki, for the AB09004 Study Group Investigators

**Affiliations:** 1 Alzheimer Research Center IM2A, Salpêtrière Hospital, AP-HP, Sorbonne University, Paris, France; 2 Cantoblanco Memory Clinic, Geriatric Department, Hospital Cantoblanco, Madrid, Spain; 3 The Dr Stanley Lipschitz Clinic Inc, Rosebank, Johannesburg, South Africa; 4 Neurological Department, Athens Naval Hospital, Athens, Greece; 5 Carol Davila University of Medicine and Pharmacy, The Excellence Clinic of Geriatrics, Gerontology and Old Age Psychiatry, Bucharest, Romania; 6 The Excellence Memory Center and Longevity Medicine, “Ana Aslan” International Foundation, Bucharest, Romania; 7 Psychosomatic Center Based On Psychoneurology Department of Communal Enterprise ‘Dnipropetrovsk Regional Clinical Hospital Named After I.I. Mechnikov’, Dnipropetrovsk Regional Council, Dnipro, Ukraine; 8 Department Psychiatry, Narcology and Medical Psychology I, Horbachevsky Ternopil National Medical University, Ternopil, Ukraine; 9 Univ. Lille, UMR Inserm U1172, CHU Lille, FHU Precise, F‑59000 Lille, France; 10 AB Science, Paris, France; 11 Imagine Institute, INSERM UMR 1163, University of Paris, Laboratory of Cellular and Molecular Mechanisms of Hematological Disorders and Therapeutic Implication, Hôpital Necker, Paris, France; 12 Department of Hematology, Necker Hospital, Assistance Publique Hôpitaux de Paris, Paris, France; 13 First Department of Neurology, School of Medicine, Faculty of Health Sciences, Aristotle University of Thessaloniki, Thessaloniki, Macedonia Greece

**Correction** **:** **Alz Res Therapy 15, 39 (2023)**

**https://doi.org/10.1186/s13195-023-01169-x**

Following publication of the original article [1], the authors identified an error in the author name of Triantafyllos Doskas. The given name and family name were reversed.

The incorrect author name is: Doskas Triantafyllos

The correct author name is: Triantafyllos Doskas

The author group has been updated above and the original article [1] has been corrected.
