# Supplementary material for: Masitinib for mild-to-moderate Alzheimer’s disease: results from a randomized, placebo-controlled, phase 3, clinical trial
Source: Alzheimers Res Ther. 2023 Feb 28;15:39. doi: 10.1186/s13195-023-01169-x (PMC9972756; doi:10.1186/s13195-023-01169-x)
Supplement: Supplementary file 1 — Additional file 1: Supplemental methods. Full Analysis Dataset (FAS) definition for primary efficacy analysis of the masitinib 4.5 mg/kg/day parallel group. eTable 1. Summary of reasons for discontinuation before week 24, based on information recorded on the case report form (CRF) end-of-study page (Safety dataset). eTable 2. Exploratory post-hoc analysis for the masitinib 4.5 mg/kg/day parallel group based on MMRM methodology (FAS population). eTable 3. Summary of most frequent severe adverse events for masitinib cohorts relative to pooled placebo cohort over the 24-week treatment period and corresponding incidence rate ratios (Safety dataset). eTable 4. Summary of most frequent non-fatal serious adverse events for masitinib cohorts relative to pooled placebo cohort over the 24-week treatment period and corresponding incidence rate ratios (Safety dataset). eTable 5. Additional pooled placebo sensitivity analysis for the titrated masitinib 6.0 mg/kg/day parallel group (FAS population). eFigure 1. Treatment effect on ADAS-cog and ADCS-ADL between week 0 and week 24 in the 4.5 mg/kg/day parallel group (left panel) and titrated 6.0 mg/kg/day parallel group (right panel). [file 13195_2023_1169_MOESM1_ESM.pdf]

Supplement to ‘Masitinib for Mild-to-Moderate Alzheimer's Disease: Results from a Randomized, Placebo-Controlled, Phase 3, Clinical Trial’

This appendix has been provided by the authors to give readers additional information.

Table of Contents

AB09004 Study Group Investigators listed by country 2

SUPPLEMENTAL METHODS 3

Full Analysis Dataset (FAS) definition for primary efficacy analysis of the masitinib 4.5 mg/kg/day parallel group 3

SUPPLEMENTAL TABLES 4

eTable 1: Summary of reasons for discontinuation before week 24, based on information recorded on the case report form (CRF) end-of-study page (Safety dataset) 4

eTable 2: Exploratory post-hoc analysis for the masitinib 4.5 mg/kg/day parallel group based on MMRM methodology (FAS population). 5

eTable 3: Summary of most frequent severe adverse events for masitinib cohorts relative to pooled placebo cohort over the 24-week treatment period and corresponding incidence rate ratios (Safety dataset) 6

eTable 4: Summary of most frequent non-fatal serious adverse events for masitinib cohorts relative to pooled placebo cohort over the 24-week treatment period and corresponding incidence rate ratios (Safety dataset) 7

eTable 5: Additional pooled placebo sensitivity analysis for the titrated masitinib 6.0 mg/kg/day parallel group (FAS population). 8

SUPPLEMENTAL FIGURES 9

eFigure 1: Treatment effect on ADAS-cog and ADCS-ADL between week 0 and week 24 in the 4.5 mg/kg/day parallel group (left panel) and titrated 6.0 mg/kg/day parallel group (right panel). 9

AB09004 Study Group Investigators listed by country

ARGENTINA
- Adriana Ellenberg
- Conrado Estol
- Janus Kremer
- Hector Lamaison
- Ignacio MacKinnon
- Hector Mauriño
- Gustavo Saredo

BULGARIA
- Plamen Georgiev
- Spaska Georgieva
- Ara Kaprelyan
- Borislav Kralev
- Veska Markova
- Rumelia Mitkova
- Maria Nikolova
- Penko Shotekov
- Slavcho Sirakov
- Nikolay Sotirov
- Deyana Staneva

FRANCE
- Bruno Dubois

GREECE
- Triantafyllo Doskas
- Nikolaos Fakas
- Xenofon Fitsioris
- Elisabeth Kapaki
- Dimitrios Kalochristianakis
- Dimitrios Mitsikostas
- Anastasios Orologas
- Paraskevi Sakka
- Antonios Tavernarakis
- Magda Tsolaki
- Ioannis Zaganas

HONG KONG
- Patrick Chiu
- Ming Chu Edith Lau

ISRAEL
- Yehudit Aharon-Perez
- Zeev Meiner

MALAYSIA
- Ai-Vyrn Chin
- Yotin Chinvarun
- Esther Ebenezer

PERU
- Carlos Cosentino
- Nilton Custodio
- Liliana Rodriguez

PHILIPPINES
- Joel Advincula
- Jacqueline Dominguez
- Artemio Roxas, Jr

POLAND
- Maciej Czarnecki
- Małgorzata Czernichowska-Kotiuszko
- Ewelina Górska
- Małgorzata Kulka
- Andrzej Szczudlik

ROMANIA
- Sanda Deme
- Mihaela Rosca
- Luiza Spiru
- Szalbocs Szatmári
- Ana Maria Tuculanu

RUSSIA
- Mikhail Sherman

SINGAPORE
- Christopher Chen
- Nagaendran Kandiah

SLOVAKIA
- Andrea Cimprichová
- Peter Čuchran
- Beata Dupejova
- Miloslav Dvorák
- Ladislav Gurčík
- Gregušová Judita
- Georgi Krastev
- Moravčík Kvetoslav
- Puzderová Liana
- Nyéky Mikuláš
- Magdalena Perichtová
- Stefan Smik
- Renáta Smiková
- Peter Turcani

SOUTH AFRICA
- Susan Arnold
- Nyda Fourie
- Judy Green
- Stanley Lipschitz
- Felix Potocnik
- Juan Schronen

SPAIN
- Pedro Abizanda
- Manuel Anton
- Carmen Antunez
- Mercé Boada
- Cristobal Carnero
- Alfonso Cruz
- Pedro Gil
- Miguel Goñi
- Jesús López-Arrieta
- Esperanza Martin
- Javier Olazaran
- Roberto Petidier
- Jose Manuel Vega-Andion
- Alberto Villarejo

TAIWAN
- Chin-Vhang Huang
- Ming-Chyi Pai
- Yuan-Han Yang
- Yu-Wan Yang

THAILAND
- Yotin Chinvarun

TUNISIA
- Mahbouba Frih-Ayed
- Riadh Gouider
- Chokri Mhiri
- Ridha Mrissa

UKRAINE
- Zhanna Bazik
- Ihor Bielyi
- Iurii Buchok
- Rostyslav Bilobryvka
- Roman Khaitov
- Inna Makarova
- Olena Moroz
- Svetlana Moroz
- Pavlo Palamarchuk
- Igor Pasyura
- Sofiya Rimsha
- Vladlena Semenikhina
- Oksana Serebrennikova
- Nataliia Turishcheva
- Olena Venger
- Anatolii Voloshchuk
- Hennadii Zil'berblat

UNITED KINGDOM
- Boben Benjamin
- Roger Bullock
- Sajeev Kshemendran
- Catherine Mummery
- Ramin Nilforooshan
- Stephen Pearson

SUPPLEMENTAL METHODS

Full Analysis Dataset (FAS) definition for primary efficacy analysis of the masitinib 4.5 mg/kg/day parallel group

Primary efficacy analysis was done according to a prospectively defined full analysis dataset (FAS) in which patients received at least one dose of study treatment (masitinib/placebo) and whose scores had not been potentially influenced by any source of strong bias during the 24-week assessment period (as determined by predefined rules and validated by the Independent Data Monitoring Committee prior to unblinding). This included patients with change of caregiver between baseline and week 8 to 24, or extreme fluctuations reported for the primary endpoints; for example, abnormally high or low baseline ADCS-ADL and/or ADAS-Cog as compared to the patients' prior status, or sudden improvement of more than 10 points at week 24 that was not substantiated by a trend of improvement at week 8 or 12 or explained by an introduction or change of care. The evaluable population was further defined by exclusion of patients that encountered critical issues including non-respect of Good Clinical Practice (GCP).

This modification to the intention to treat (ITT) population was considered necessary because the inclusion of such patients could confound interpretation of the results. Nevertheless, ITT analysis was performed as a sensitivity analysis.

Considering the FAS for the masitinib 4.5 mg/kg/day parallel group, 12 patients were excluded from its associated ITT population (3 and 9 from the masitinib and placebo treatment-arms, respectively) for the following reasons:

a) Two (2) patients received no treatment intake
b) One (1) patient did not have Alzheimer's disease (documented letter from the investigator)
c) Three (3) patients had baseline ADCS-ADL and/or ADAS-Cog scores that did not correspond to the medical history (documented letters from the investigators)
d) Two (2) patients with change of caregiver during the 24-week assessment period (documented letters from the investigators).
e) Five (5) patients with critical GCP violations (highlighted by audit and internal reports).

SUPPLEMENTAL TABLES

eTable 1: Summary of reasons for discontinuation before week 24, based on information recorded on the case report form (CRF) end-of-study page (Safety dataset)

	M3.0 (N=58)	M4.5 (N=185)	tM6.0 (N=186)	Pooled PBO (N=280)
**Total Discontinuations, n (%)**	**14 (24.1)**	**58 (31.4)**	**76 (40.9)**	**41 (14.6)**
**Reasons:**				
TEAE (non-fatal) related to study treatment	7 (12.1)	23 (12.4)	40 (21.5)	11 (3.9)
Death (under treatment + 24 h)	0	1 (0.5)	1 (0.5)	0
Lack of efficacy	0	1 (0.5)	2 (1.1)	5 (1.8)
Caregiver unavailability	1 (1.7)	2 (1.1)	3 (1.6)	0
Compliance to IMP and protocol procedures	0	0	0	1 (0.4)
TEAE (not related to study treatment) leading to death	0	0	0	1 (0.4)
Family reasons	1 (1.7)	1 (0.5)	2 (1.1)	0
Use of prohibited/forbidden concomitant treatment	0	2 (1.1)	1 (0.5)	1 (0.4)
Inclusion/exclusion criteria violation	0	2 (1.1)	4 (2.2)	4 (1.4)
Lost to follow-up	0	2 (1.1)	1 (0.5)	2 (0.7)
Patient request (related to procedures/travel)	4 (6.9)	12 (6.5)	11 (5.9)	6 (2.1)
Patient moved to another town	0	3 (1.6)	8 (4.3)	4 (1.4)
Reason unknown	0	4 (2.1)	0	1 (0.4)
TEAE (non-fatal) not related to study treatment	1 (1.7)	5 (2.7)	3 (1.6)	5 (1.8)

M3.0: Masitinib treatment-arm from masitinib 3.0 mg/kg/day parallel group. M4.5: Masitinib treatment-arm from masitinib 4.5 mg/kg/day parallel group. tM6.0: Masitinib treatment-arm from titrated 6.0 mg/kg/day parallel group. PBO: Placebo. TEAE: Treatment-Emergent Adverse Events. IMP: Investigational Medicinal Product.

eTable 2: Exploratory post-hoc analysis for the masitinib 4.5 mg/kg/day parallel group based on MMRM methodology (FAS population).

M4.5 parallel group		PBO (N=176)	M4.5 (N=182)	Between group difference	P value
	**Primary analysis**	**Change from baseline at week-24 (±SE)**		**LSM (97.5% CI)**	
	ADAS-Cog (primary endpoint)	0.69 (±0.54)	-1.46 (±0.51)	-2.15 [-3.48, -0.81]	<0.001
	ADCS-ADL (primary endpoint)	-0.81 (±0.79)	1.01 (±0.76)	1.82 [-0.15, 3.79]	0.038
	**Exploratory analysis**	**Change from baseline at week-24 (±SE)**		**LSM (95% CI)**	
	ADAS-Cog (MMRM post-hoc)	-0.19 (±0.36)	-1.14 (±0.37)	-0.95 [-1.89, -0.02]	0.046
	ADCS-ADL (MMRM post-hoc)	0.17 (±0.54)	0.81 (±0.56)	0.64 [-0.82, 2.10]	0.390

MMRM: mixed model for repeated measures. FAS: full analysis dataset. M4.5: Masitinib treatment-arm from the masitinib 4.5 mg/kg/day parallel group. PBO: Placebo treatment-arm from the masitinib 4.5 mg/kg/day parallel group. LSM: Least-squares mean. ADAS-cog: Alzheimer's Disease Assessment Scale. For ADAS-cog (scores range from 0 to 70) a positive change from baseline indicates worsening dementia and a negative between group difference (masitinib minus placebo) favors masitinib. ADCS-ADL: Alzheimer's Disease Cooperative Study Activities of Daily Living Inventory scale. For ADCS-ADL (scores range from 0 to 78) a negative change from baseline indicates worsening function and a positive between group difference (masitinib minus placebo) favors masitinib. SE: Standard error. CI: Confidence Interval at 97.5% for primary endpoint and at 95% for sensitivity analyses.

MMRM post-hoc analysis based on the primary endpoints of ADAS-cog score until week 24 and ADCS-ADL score until week 24, was preformed using observed data alone (i.e., without any kind of imputation) in the full analysis dataset (placebo n=176; masitinib n=182), including data at weeks 8, 12 and 24 (i.e., repeated measures). For analysis of ADAS-cog, the following covariates were used: treatment group, time point, age, MMSE (categorical, 21–25 versus 12–20), and baseline ADAS-cog. For analysis of ADCS-ADL, the following covariates were used: treatment group, time point, age, MMSE (categorical, 21–25 versus 12–20), baseline ADCS-ADL and baseline ADAS-cog. It is noted that this model is equivalent to that predefined for analysis of secondary endpoints.

Results showed that masitinib (4.5 mg/kg/day) has a statistically significant benefit relative to placebo over 24 weeks on the endpoint of ADAS-cog, with a δADAS-cog of -1.14 versus -0.19, respectively, and corresponding ΔADAS-cog of -0.95 (95%CI [-1.89, -0.02]) (a negative value favors masitinib); p=0.046. The post-hoc MMRM analysis of ADCS-ADL over 24 weeks showed a masitinib δADCS-ADL of +0.80 (representing an overall functional improvement) versus a baseline change of +0.17 for placebo, giving a nonsignificant ΔADCS-ADL of +0.64 (95%CI [(-0.82, 2.10]); p=0.39.

eTable 3: Summary of most frequent severe adverse events for masitinib cohorts relative to pooled placebo cohort over the 24-week treatment period and corresponding incidence rate ratios (Safety dataset)

Pts with ≥1 event; % (n)	**M3.0 (N=58)**	**$IRR_{[M3.0]}$**	**M4.5 (N=185)**	**$IRR_{[M4.5]}$**	**tM6.0 (N=186)**	**$IRR_{[tM6.0]}$**	**Pooled PBO (N=280)**
**Neutropenia**	5.2% (3)	4.7	2.7% (5)	2.5	3.8% (7)	3.5	1.1% (3)
**Neutrophil count decreased**	1.7% (1)	1.5	1.1% (2)	1.0	2.2% (4)	2.0	1.1% (3)
**GGT increased**	0	0	1.6% (3)	2.3	1.6% (3)	2.3	0.7% (2)
**White blood cell count decreased**	0	0	0.5% (1)	1.3	1.6% (3)	4.0	0.4% (1)
**Hypophosphatemia**	0	0	0	0	1.1% (2)	2.8	0.4% (1)
**Lymphocyte count decreased**	1.7% (1)	2.4	1.6% (3)	2.3	1.1% (2)	1.6	0.7% (2)
**Lymphopenia**	0	N/A	1.1% (2)	N/A	1.1% (2)	N/A	0
**Stevens-Johnson syndrome**	0	N/A	0	N/A	1.1% (2)	N/A	0
**Weight decreased**	0	N/A	0	N/A	1.1% (2)	N/A	0
**Blood potassium decreased**	0	0	1.6% (3)	4.0	0.5% (1)	1.3	0.4% (1)
**AST increased**	0	0	1.1% (2)	2.8	0.5% (1)	1.3	0.4% (1)
**Blood phosphorus decreased**	0	0	3.8% (7)	9.5	0.5% (1)	1.3	0.4% (1)
**Renal failure**	0	N/A	1.1% (2)	N/A	0.5% (1)	N/A	0
**Diarrhea**	0	N/A	1.1% (2)	N/A	0	N/A	0
**Sepsis**	0	N/A	1.1% (2)	N/A	0	N/A	0

Severe adverse events (grades 3 or 4) that occurred in at least 2 patients are listed (ordered according difference in incidence between the titrated masitinib 6.0 mg/kg/day and pooled placebo cohorts). Adverse event (AE) described using MedDRA preferred terms. AEs were recorded until 28 days after treatment interruption. Any given AE can be listed under multiple MedDRA preferred terms, which are not therefore cumulative. M3.0: Masitinib treatment-arm from masitinib 3.0 mg/kg/day parallel group. M4.5: Masitinib treatment-arm from masitinib 4.5 mg/kg/day parallel group. tM6.0: Masitinib treatment-arm from titrated 6.0 mg/kg/day parallel group. PBO: Placebo. IRR[M/P]: Incidence rate ratio for given masitinib cohort as compared with pooled placebo cohort (M/P). GGT: Gamma-glutamyltransferase. AST: Aspartate aminotransferase. N/A: Not applicable.

eTable 4: Summary of most frequent non-fatal serious adverse events for masitinib cohorts relative to pooled placebo cohort over the 24-week treatment period and corresponding incidence rate ratios (Safety dataset)

Pts with ≥1 event; % (n)	**M3.0 (N=58)**	**$IRR_{[M3.0]}$**	**M4.5 (N=185)**	**$IRR_{[M4.5]}$**	**tM6.0 (N=186)**	**$IRR_{[tM6.0]}$**	**Pooled PBO (N=280)**
**Neutropenia**	5.2% (3)	N/A	1.6% (3)	N/A	1.6% (3)	N/A	0
**Stevens-Johnson syndrome**	0	N/A	0	N/A	1.6% (3)	N/A	0
**Neutrophil count decreased**	0	N/A	0	N/A	1.1% (2)	N/A	0
**Edema peripheral**	0	N/A	0	N/A	1.1% (2)	N/A	0
**Pneumonia**	0	0.00	1.6% (3)	4.00	0.5% (1)	1.25	0.4% (1)
**Renal failure**	0	N/A	1.1% (2)	N/A	0	N/A	0
**Sepsis**	0	N/A	1.1% (2)	N/A	0	N/A	0

Non-fatal serious adverse events (SAE) that occurred in at least 2 patients are listed (ordered according difference in incidence between the titrated masitinib 6.0 mg/kg/day and pooled placebo cohorts). SAEs described using MedDRA preferred terms. SAEs were recorded until 28 days after treatment interruption. Any given SAE can be listed under multiple MedDRA preferred terms, which are not therefore cumulative. M3.0: Masitinib treatment-arm from masitinib 3.0 mg/kg/day parallel group. M4.5: Masitinib treatment-arm from masitinib 4.5 mg/kg/day parallel group. tM6.0: Masitinib treatment-arm from titrated 6.0 mg/kg/day parallel group. PBO: Placebo. IRR[M/P]: Incidence rate ratio for given masitinib cohort as compared with pooled placebo cohort (M/P). N/A: Not applicable.

eTable 5: Additional pooled placebo sensitivity analysis for the titrated masitinib 6.0 mg/kg/day parallel group (FAS population).

	**Change from baseline at week-24 (±SE)**		**Least-squares mean difference**	**P value**
	**Pooled PBO (N=267)**	**tM6.0 (N=186)**		
ADAS-Cog (pooled placebo sensitivity analysis)	0.87 (±0.41)	-0.07 (±0.45)	- 0.94 (95% CI [-1.88, -0.001])	0.0497
ADCS-ADL (pooled placebo sensitivity analysis)	-0.65 (±0.61)	0.47 (±0.67)	1.12 (95% CI [(-0.28, 2.52])	0.118

This table summarizes data from patients in the full analysis dataset (FAS). ADAS-cog: Alzheimer's Disease Assessment Scale. For ADAS-cog (scores range from 0 to 70) a positive change from baseline indicates worsening dementia and a negative between group difference (masitinib minus placebo) favors masitinib. ADCS-ADL: Alzheimer's Disease Cooperative Study Activities of Daily Living Inventory scale. For ADCS-ADL (scores range from 0 to 78) a negative change from baseline indicates worsening function and a positive between group difference (masitinib minus placebo) favors masitinib. SE: Standard error. CI: Confidence Interval at 95% for sensitivity analyses.

Results from the titrated masitinib 6.0 mg/kg/day parallel group did not demonstrate any treatment effect. One explanation of this divergent result is that the titrated 6.0 mg/kg/day parallel group placebo arm showed an atypical improvement over 24 weeks, as exemplified by the positive change from baseline in ADCS-ADL score. This scenario is supported by additional sensitivity analysis using the pooled placebo cohort (n=267), in which worsening in ADAS-cog and ADCS-ADL is observed for placebo, as is to be expected, with stable or improved score for the titrated masitinib 6.0 mg/kg/day cohort.

SUPPLEMENTAL FIGURES

eFigure 1: Treatment effect on ADAS-cog and ADCS-ADL between week 0 and week 24 in the 4.5 mg/kg/day parallel group (left panel) and titrated 6.0 mg/kg/day parallel group (right panel).

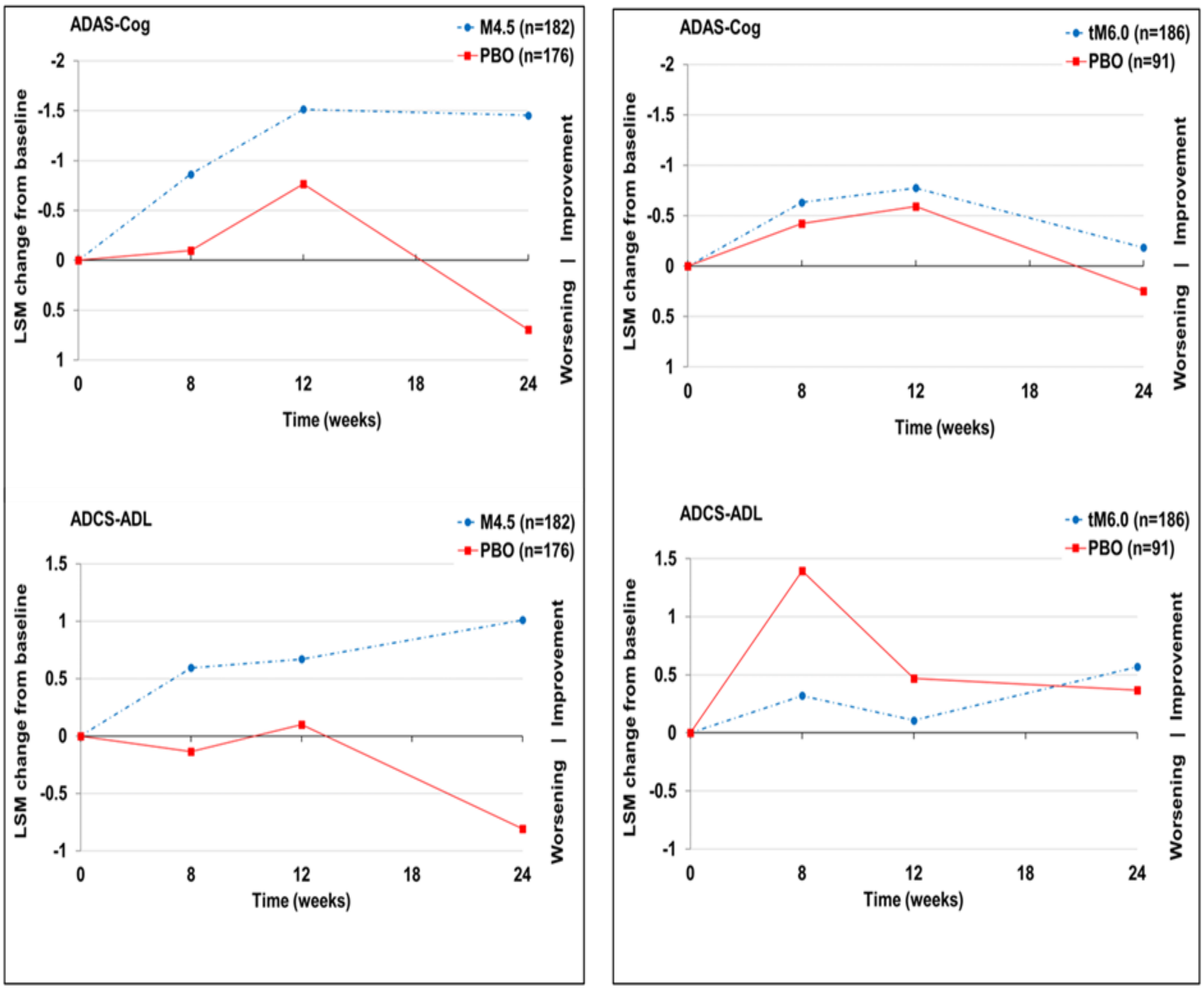

ADAS-cog: Alzheimer's Disease Assessment Scale. For ADAS-cog, a positive change from baseline indicates worsening dementia. ADCS-ADL: Alzheimer's Disease Cooperative Study Activities of Daily Living Inventory scale. For ADCS-ADL, a negative change from baseline indicates worsening function. M4.5: Masitinib treatment-arm from masitinib 4.5 mg/kg/day parallel group. tM6.0: Masitinib treatment-arm from titrated 6.0 mg/kg/day parallel group. PBO: Placebo.

These timeseries show that over the 24-week treatment period, masitinib 4.5 mg/kg/day maintained an overall improvement relative to baseline in cognition (according to ADAS-cog) and also functional improvement (according to ADCS-ADL), whereas there was an overall increased cognitive and functional deterioration in the placebo arm at week 24 (left panels). An overall improvement relative to baseline in cognitive and functional improvement was also seen for titrated masitinib 6.0 mg/kg/day, albeit to a lesser degree than for the masitinib 4.5 mg/kg/day parallel group, however, the placebo arm of the titrated 6.0 mg/kg/day parallel group showed atypical improvement over 24 weeks, as exemplified by the positive change from baseline in ADCS-ADL score (lower right panel).
